# A Qualitative Investigation of Health Care Professionals’, Patients’ and Partners’ Views on Psychosocial Issues and Related Interventions for Couples Coping with Cancer

**DOI:** 10.1371/journal.pone.0133837

**Published:** 2015-07-29

**Authors:** Tim Regan, Janelle V. Levesque, Sylvie D. Lambert, Brian Kelly

**Affiliations:** 1 Health Behaviour Research Group, Faculty of Health, School of Medicine and Public Health, The University of Newcastle, Newcastle, NSW, Australia; 2 Centre for Oncology Education and Research Translation (CONCERT)–Psycho-Oncology, Ingham Institute for Applied Medical Research, South Western Sydney Clinical School, UNSW Medicine, The University of New South Wales, Sydney, NSW, Australia; 3 Ingram School of Nursing, McGill University, Montreal, Quebec, Canada; 4 Centre for Translational Neuroscience and Mental Health, School of Medicine and Public Health, Faculty of Health and Medicine, The University of Newcastle, Newcastle, NSW, Australia; Queensland University of Technology, AUSTRALIA

## Abstract

**Introduction:**

There is growing evidence that cancer affects couples as an interdependent system and that couple-based psychosocial interventions are efficacious in reducing distress and improving coping skills. However, adoption of a couples-focused approach into cancer care is limited. Previous research has shown that patients and partners hold differing views from health care professionals (HCPs) regarding their psychosocial needs, and HCPs from different disciplines also hold divergent views regarding couples’ psychosocial needs. This study aimed to explore the perspectives of HCPs and couples on the provision of couple-focused psychosocial care in routine cancer services.

**Methods:**

A qualitative study using semi-structured interviews was undertaken with 20 HCPs (medical oncologists, nurses, psycho-oncology professionals) and 20 couples where one member had been diagnosed with cancer (breast, prostate, head/neck, bowel, multiple myeloma). Interviews were analysed using the framework approach.

**Results:**

Three core themes were identified: “How Do Couples Cope with Cancer?” emphasised the positive and negative coping strategies used by couples, and highlighted that partners perceived a lack of engagement by HCPs. “What Is Couple-focused Psychosocial Care for People with Cancer?” described varying perspectives regarding the value of couple-focused psychosocial care and variation in the types of support couples need among HCPs and couples. Whereas most couples did not perceive a need for specialist couple-focused support and interventions, most HCPs felt couple-focused psychosocial care was necessary. “How Can Couple-Focused Psychosocial Care be Improved?” described couples’ view of a need for better provision of information, and the importance of their relationship with oncology clinicians. HCPs identified a lack of confidence in responding to the emotional needs of couples, and barriers to providing psychosocial care, including challenges identifying distress (through screening) and referring distressed individuals/couples for specialist assessment.

**Conclusions:**

The three core themes revealed discrepancies about couple-focused psychosocial care between HCPs and couples, and HCPs from different professional backgrounds, and several barriers to the provision of psychosocial care for couples. Despite HCPs and couples acknowledging that a couple-focused approach to psycho-social support was potentially beneficial, the majority of couples did not feel they needed specific couple-focused interventions. These issues and recommendations for future research are discussed.

## Introduction

There is a substantial body of evidence to support the efficacy of psychosocial interventions for people with cancer [1], including couple-based interventions [2]. Such interventions can improve a couple’s ability to adapt to the psychosocial consequences of cancer, ameliorate psychological distress, maintain or enhance quality of life (QoL) and improve couple’s communication [2–7]. These studies provide support for the inclusion of couple-based psychosocial interventions within routine cancer care [8, 9]. Nevertheless, despite a strong focus on improving the availability and delivery of these interventions [9–12], progress in translating this evidence in to routine care is slow [8].

Both couple and health care professional (HCP) related barriers have been identified to prevent the implementation of couple-based interventions in routine cancer care. From the couples’ perspective, a recent systematic review found overall low uptake (48.8%) of couple-based interventions and high attrition rates (25.7%) [13]. Interventions requiring couples to participate simultaneously or to travel presented considerable difficulty for potential participants. Other known barriers to the uptake of psychosocial interventions by patients and their partners include having negative views regarding psychosocial support [14] and financial concerns regarding utilising these services [15]. Additional studies exploring patients’ and partners’ views of the delivery of psychosocial care have shown that although patients are typically satisfied with their psychosocial care, partners are often less so [16–19]. Often this dissatisfaction stems from the poor provision of information regarding cancer treatment, including the physical and emotional side-effects [17–19], a perceived lack of understanding of patients’ and partners’ psychosocial issues [16, 19], and a lack of support for partners and/or family [16–18]. The lack of support for partners and families is of particular concern, given partners of patients with cancer take on the majority of caregiving responsibilities [20], and often report a greater number of unmet needs [20, 21] and anxiety [22, 23] than patients.

A number of studies have broadly explored HCPs’ views on the implementation of psychosocial care [16, 24–29]. However, few studies have specifically explored HCP related barriers to the implementation of couple-based interventions in routine cancer care [13, 30]. Across studies, attitudes among HCPs’ about psychosocial care of patients with cancer seem to be a salient barrier [16, 24–29]. Current clinical practices guidelines encourage HCPs to screen for distress using specific screening questions and tools [10, 31]. However, across disciplines, HCPs have been found to have divergent views about the importance of identifying and addressing psychosocial issues [24, 26, 28] and who should provide psychosocial support [25]. Discrepancies also exist between HCPs and couples [26, 27, 32, 33]. For instance, studies have shown that, in comparison to patients and partners, HCPs often underestimate the importance of couples’ psychosocial concerns [26], their communication and spirituality needs [33], and sexuality needs [32], and patients’ ability to cope with the ongoing cancer-related issues (e.g., symptom management, seeking support) [27]. These conflicting perspectives and attitudes among HCPs might negatively impact on patients’ and caregivers’ experience, and the HCPs providing care in general [34, 35].

Although the attitudes and beliefs of HCPs, patients and their partners impact on the acceptability and dissemination of psychosocial support in routine practice [10], little is known about the barriers that influence implementation of psychosocial support for couples, as opposed to individuals. The current study addressed this research gap by taking a broader view of couple-focused psychosocial support, and explored the couples’ views regarding their psychosocial needs from a dyadic perspective, and the views held by HCPs regarding the role of couples-focused psychosocial support in cancer care. Exploring these issues among patients, partners and HCPs enables investigation of areas of consensus and divergence. The aims of this study were to explore and contrast HCPs’ and couples’ perspectives on the psychosocial issues couples face, the role of psychosocial interventions, and what barriers may interfere with the uptake of evidence-based couple-focused interventions in routine care.

## Methods

Ethical approval was obtained to conduct this study from the Hunter-New England Human Research Ethics Committee, approval number 11/09/21/5.06 (ratified by the University of Newcastle Human Research Ethics Committee). Participants provided written consent prior to participating in the study.

This report is primary analysis of qualitative data collected via 1:1 interviews with health care professionals and 1:2 interviews with patients and their partners. The Framework approach was chosen to help guide data analysis [36, 37]. This method was selected as it driven by of participants’ perspectives thus requiring a thorough review of the data. The Framework approach outlines five interconnected stages of systematic analysis that results in a clear audit trail, encouraging greater analytical rigour [38, 39].

### Sample and procedures

Convenience samples of oncology-based HCPs and couples were recruited between March 2012 and October 2013 from regional area of New South Wales, Australia. The HCPs included medical oncologists, nurses, social workers and psycho-oncology professionals. HCPs were contacted by email and invited to participate in a 30-minute telephone interview. A consent form, information statement, and a demographic survey were then e-mailed to those who responded favourably to this initial invitation. The main inclusion criteria were: fluent in English, experience in providing supportive care to couples facing a cancer diagnosis, and an interest in discussing their views on the psychosocial care of couples. As patterns in the findings emerged, purposeful sampling of HCPs was undertaken [40]. For example, cancer care nurses were often identified by other HCPs as being key to the psychosocial care of couples, and therefore more were recruited to enhance variability and comparability within the sample. Fifty-six health care professionals were contacted, with 22 responding to the study invitation and of these, 20 participated in an interview. Specific reasons for refusal were difficult to obtain as non-responders to email invitations were not followed-up (as per ethics agreement). Non-participation by two HCPs after originally agreeing was due to participants not being able to participate in the scheduled interview, and not responding to follow-up calls. Table 1 summarises the professional characteristics of the participating HCPs.

**Table 1:** HCPs’ Professional Experience.

	Participants (n = 20)
**Gender, N(%)**	
Male	8 (40)
Female	12 (60)
**Profession, N(%)**	
Oncologist	7 (35)
Psychologist/psychiatrist	6 (30)
Nursing	5 (25)
Social worker	2 (10)
**No. Years Professional Experience, N(%)**	
0–5	0
5–10	4 (20)
More than 10	16 (80)
**No. Years Cancer Experience, N(%)**	
0–5	3 (15)
5–10	4 (20)
More than 10	13 (65)
**Health Service Location, N(%)**	
Metropolitan	10 (50)
Regional	10 (50)

Couples (i.e., two adults in an intimate relationship) were recruited from support groups, psycho-oncology services, and hospital-based oncological services. Several recruitment strategies were used: 1) Members of the research team attended support group meetings, introduced the study to the group, and provided interested individuals or couples with a study pack. Verbal consent was obtained at this time to contact interested parties by telephone to discuss the study further; 2) Members of the research team attended multi-disciplinary clinics, and were introduced to potential participants by clinic staff. The study was introduced, study packs were provided to interested parties, and verbal consent was obtained to make contact by phone to discuss the study further; 3) Study packs were provided to clinical staff, who passed them on directly to potential participants. Clinical staff obtained verbal consent from participants for members of the research team to follow-up with couples by phone, to discuss the study further. Four couples were referred through other studies conducted by the authors. The main inclusion criteria were: fluent in English, at least one member had received a cancer diagnosis, and both members of the couple were interested in discussing their experiences. Interviews were not scheduled until written consent had been received. One hundred and seven eligible patients were initially invited to participate in the study. If their partner was also present, they were directly invited as well. If not, with patients’ permission, they were contacted by phone and invited. Twenty-two patients and their partners agreed to be interviewed, and 20 completed the interview, resulting in a consent rate of 20.5%. The two most common reasons for declining study invitation were lack of interest (n = 24) or a lack of time (n = 10). Reasons for refusal could not be obtained for 51 couples, as they were provided study packs by clinic staff and ethics approval precluded follow-up with non-responders. Two couples did not complete the interview after providing consent due to the death of the patient (n = 1), and lack of interest (n = 1). Table 2 summarises the demographic characteristics of the couples interviewed. It should be noted that recruitment was open to couples of all sexual orientations no same-sex couples participated.

**Table 2:** Couple Demographics.

	Participants (n = 20)
**Patient gender, N (%)**	
Male	13 (65)
Female	7 (35)
**Age (years), M (SD)**	
Patient	64.6 (9)
Partner	63.5 (9.1)
**Relationship length (years), M (SD)**	36.8 (14.9)
**Cancer diagnosis, N (%)**	
Prostate	8 (40)
Breast	6 (30)
Head & neck	4 (20)
Multiple myeloma	1 (5)
Bowel	1 (5)
**Time since diagnosis (months), M (SD)**	14.4 (17.6)

### Data collection

#### Semi-structured interviews

Semi-structured interviews were conducted with all participants. For HCPs, 10 face-to-face interviews were conducted in private rooms at two separate hospital sites in New South Wales, Australia. The remaining 10 interviews were completed over the phone. The majority of couples were interviewed in their homes (n = 18). Two telephone interviews were conducted with couples. All interviews were conducted by the first author (TR). The interviewer had established relationships with some HCPs prior to commencing the study to the extent that they are part of a common professional network. For couples, only cancer type, treatments, and some basic demographic details were known prior to the interviews. All participants were interviewed once, and interview length ranged from 12 to 40 minutes (*M* = 27 minutes, SD = 9 minutes) for HCPs, and from 19 to 79 minutes (M = 47 minutes, SD = 18 minutes) for couples. All interviews were audio-recorded and transcribed verbatim with transcripts sent to participants who requested them. Table 3 outlines the interview guide used to focus the discussion. The interview guide was developed to be consistent with elements of the Institute of Medicine’s Patient-Centred Care framework, and in particular, its focus on the involvement of families and support **persons** [41]. The interview guide was used flexibly with respect to the order of questions and the depth to which some issues were explored.

**Table 3:** Examples of interview questions.

HCPs		Couples	
Interview stage	Examples of questions	Interview stage	Examples of questions
*STAGE 1*: Overall impression of challenges faced by couples	*‘In your experience*, *what are some of the biggest challenges couples face following a cancer diagnosis*?*’*	*STAGE 1*: Response to cancer diagnosis and treatment	‘*How did you respond to the diagnosis*?*”*
*STAGE 2*: Your role in facilitating the needs of patient, as well as the needs of the partner	‘*What role do you play in assisting the couple manage distress*?	*STAGE 2*: Nature of psychosocial support received following diagnosis	*‘Can you describe how you and your spouse coped during the treatment*?*’*
*STAGE 3*: Opinions about how health services could be improved to better assist couples following a cancer diagnosis	*‘How do you feel our health services can better assist couples*?	*STAGE 3*: Opinions needs of couples following a cancer diagnosis	*‘What could have been improved to ensure you had all the support you needed*?*’*

#### Data analysis

Transcripts were analysed by hand to highlight initial themes before all interviews were entered into NVivo 10 to further develop the thematic framework. All of the transcripts were coded by one author (TR), one author coded six couple and six HCP transcripts (JL) and one other co-author (BK) coded six HCP transcripts. All authors contributed to data analysis and refinement of coding. Interviewer and analytical biases were managed during regular analysis meetings among all authors. The first and second authors (TR, JL) engaged in regular discussion of cases throughout the data analysis phase to ensure rigor. In regards to the themes that were identified, a high degree of similarity was found among the authors and all agreed with the revisions made to the interpretative framework.

The framework approach outlines five stages of data analysis: familiarisation with the data, identification of thematic framework (using a priori aims, and issues that emerge from the data), indexing (using the thematic framework to code the data), charting (grouping the data according to the parts of the thematic framework they correspond with using codes), and mapping and interpretation (providing explanations of the findings, and the relationships between themes as they relate to the overall aims of the study) [37]. An initial framework was developed based on the study aims and the stages of the interview process (Tables 4 & 5, column 1). The data were then indexed based on this initial framework by assigning codes to sentences and/or paragraphs that reflected the study aims. Codes were single words or short phrases that captured the essence of the excerpts. These codes were then used to revise the initial framework (Tables 4 & 5, column 2). Across interviews the dominant themes and sub-themes were identified (Tables 4 & 5, columns 3 & 4), and then compared across HCPs’ and couples’ interviews.

**Table 4:** Stages of framework analysis for HCPs’ views on psychosocial during cancer.

Initial framework based on a priori issues	Revised framework based on indexing	Themes	Sub-themes
Challenges couples face following a cancer diagnosis (emotional impact, change in relationship, financial impact, career impact, interruption to future plans, caring for patients, impact on family, impact on social life)	Major challenges faced by couples (increased burden on partner, partner as a caregiver, change in partner’s role, partner distress)	‘The Partner’s Place in Cancer Care’	Adapting to new roles; Partner as a negative influence on patient and care; Partner as ally to HCP and PT
HCPs’ role in assisting couples manage distress (providing cancer information, emotional support, involvement in couples issues, providing support to patient, providing support to partner, use of available services and resources)	HCPs’ role in assisting couples manage distress (provision of appropriate psychological support, dealing with issues not trained for, information provision)	‘Psychosocial care for one or two?’	HCPs’ Approach to Supporting Couples; Who should receive psychosocial support?; What kind of support do people need?, The Value of Psychosocial Care
Improving health services for couples (improving referrals, acknowledging couples’ issues, screening for distress, providing couple-based psychosocial therapies, improving all HCPs ability to provide support)	Improving health services (opportunities for counselling, identifying distressed couples, improving distress screening practices, resources in rural areas, acknowledging partner distres, HCP communication)	‘Issues in Distress Screening for Couples’	*Who* should be screened*; What* should we use to screen (instrument vs. experience); *When* should screen—once vs. multiple time points*; Why* should we screen at all (e.g. to tailor for interventions)
		‘The quest for adequate psychosocial care’	Barriers to adequate psychosocial care; Improving psychosocial care

**Table 5:** Stages of framework analysis for couples’ views on their experience with cancer and HCPs.

Initial framework based on a priori issues	Revised framework based on indexing	Themes	Sub-themes
Response to cancer diagnosis (emotional response, relationship impact, change in relationship roles, coping strategies, family impact, career impact, interruption to future plans, impact on social life).	Emotional response to diagnosis (role of partner, discussing cancer with family, impact on relationship/future plans/intimacy, coping strategies)	‘Responding To Cancer’	Patient responses/Partner responses/Discussing with family
		*‘*The way we cope with cancer’	Individual coping strategies, Dyadic coping strategies (emotional support, practical support, communication)
Support received following diagnosis (family, social network, HCPs, informational support, emotional support, practical support)	Support from HCPs (encouraged to seek psychosocial support, counseling, information provision, confidence in treatment, coordination of care); Support groups (emotion & practical support, shared experience)	‘Our experiences with HCPs’	Confidence in diagnosis and treatment; Provision of information; Support groups; Counselling
Views on the needs of couples following diagnosis/treatment (support for partner, improving information provision, access to psychosocial support)	Emotional response to treatment (experience of loss, body image concerns, survivor guilt); Acknowledgment of relationship (impact on partners, support for partners); Provision of cancer information (what do couples understand?, managing disparity in understanding); Follow-up care (access to psychosocial services, discussing psychosocial impact with HCPs, long-term side-effects)	Transition to Survivorship’	Managing on-going effects; Improving psychosocial support during survivorship

#### Determining sample size

Data appeared to become redundant (i.e., thematic saturation) following the 18^th^ interview for HCPs and the 17^th^ interview for couples. All authors agreed that no significant new themes were emerging within the data and that point of saturation had been reached. As participants had agreed to participate, two more interviews with HCPs and three more with couples were conducted beyond this point and included in the analysis.

### Maintaining research quality

The Consolidated Criteria for Reporting Qualitative Research framework was used to guide the reporting of the findings [42]. In addition, criteria of credibility, transferability, and confirmability were used to ensure the rigor of this study [43–48]. Strategies used to address credibility included recording interviews and transcribing them [36], authors frequently discussing findings [43, 49], encouraging participants to pursue their own lines of thinking [36], and searching the data for conflicting patterns [46]. Transferability was addressed by relating our findings to similar findings in the literature [44], clearly describing the sample and setting for this study [44, 50], and using direct quotes [48]. Confirmability was addressed by rigorous review of interview transcripts, the codes used to identify themes, and drafts and revisions of the findings [36].

## Results

**Fig 1:**
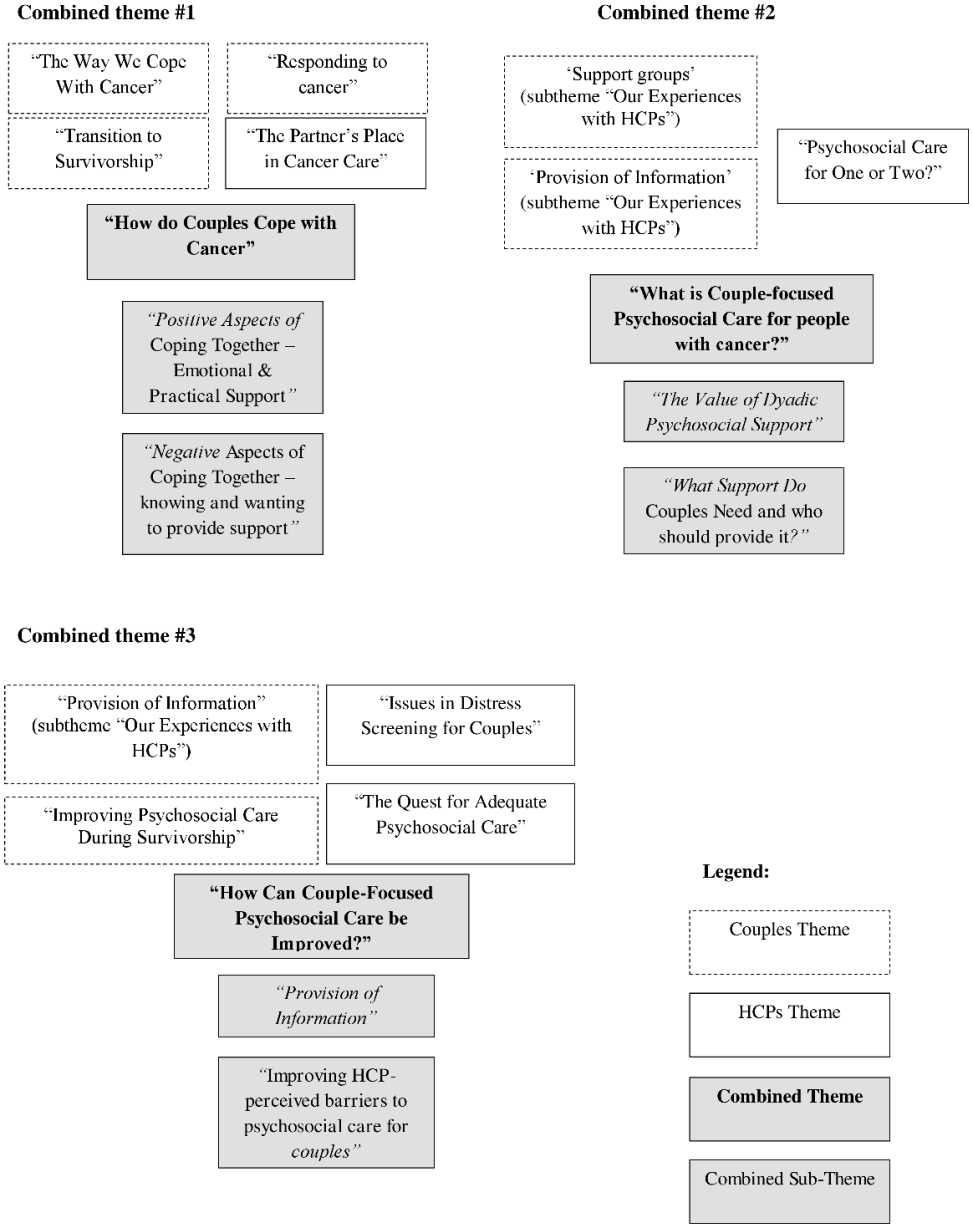
Integration of couple and HCP themes (themes from Tables 4 & 5).

Four themes emerged from the interviews with HCPs, and four from those with couples (see Tables 4 & 5, third column). HCPs’ and couples’ themes were then reviewed and similar content between the two groups were integrated resulting in three core themes (see Fig 1).

In Fig 1, couple themes are represented with dotted-line boxes, HCP themes by solid-line boxes, combined themes are in grey and bold, and combined subthemes are grey and italicised.

### How Do Couples Cope With Cancer?

“How Do Couples Cope With Cancer?” is a theme that combines the couples’ themes “The Way We Cope with Cancer”, “Responding to Cancer”, and “Transition to Survivorship”, with the HCPs’ theme “The Partners Place in Cancer Care”. Within the theme of “How Do Couples Cope With Cancer?”, two sub-themes were identified, “Positive Aspects of Coping Together” and “Negative Aspects of Coping Together”.

Although both patients and partners spoke of having to cope with a range of emotions following a cancer diagnosis, including fear, anger, and shock, a sense of helplessness was particularly evident for partners.

*“It’s a feeling of helplessness not being able to do anything for [patient]… just hoping that you put trust in the medical profession and hope they get it right*.*”*

*(Partner*, *woman with breast cancer)*

HCPs expressed similar views regarding partners’ experiences. Some HCPs suggested that partners are still grappling with their own emotional distress when they become caregivers and have to assist with new caring tasks. Further to that, HCPs also described partners’ struggle in determining how best to support the patient and reconciling their own needs for support as the ‘healthy’ person.

*“I think it’s fair to say that partners feel really lost about what support they should give… what’s their role*, *should [they] be the care co-ordinator*, *should they be a recipient of care just like the affected person is*, *should they be the counsellor or should they be the receiver of counselling*?*”*

*(Oncologist)*

HCPs, patients, and partners all described examples of helpful or adaptive ways of coping with cancer together, but only HCPs described detrimental coping responses among couples. The two aspects of helpful or adaptive partner support that were commonly identified by HCPs and couples included emotional and practical support.

The partner’s role in the provision of emotional support to the patient was most often described in terms of listening to the patient and conveying to the patient an understanding of the diagnosis and treatment. This form of support was re-iterated by couples, although patients often described partner emotional support as simply *“just being there”*. This was typified by the acknowledgement and validation of patients’ distress. In addition, patients often described emotional support as providing a sense of unity, and reinforced the notion that they were *“in this together”*.

*“Just being there is a help…that someone understands*, *appreciates what you’re going through…advice isn’t what you need so much from your partner*, *it’s just being there and understanding*.*”*

*(Patient*, *breast cancer)*

Patients, partners, and HCPs described examples of practical support such as picking up medication, arranging transport, and being present at appointments to help retain and understand information. In addition to discussing the central role of partners in providing support, HCPs further acknowledged the confidence and ability that partners needed to undertake these roles. Psycho-oncology professionals most often emphasised the communication skills couples needed to recognise each other’s distress, and be able to maintain open communication with one another. Oncologists tended to described skills pertaining to health literacy, including finding, retaining, and acting upon cancer information.

*“…the spouse or family member being there helps in retaining information*, *usually more objectively than the patient themselves*. *And it also provides me an idea of the kind of problems that the patient and the family will face depending on how they interact with one another…”*

*(Oncologist)*

Nurses and social workers focused on communication and practical tasks (e.g., managing appointment schedules, getting medication). In contrast, partners did not typically discuss whether they had specific confidence or ability to provide this support, but often mentioned that they just “got on with it”, and did the best they could at the time. HCPs also felt that they themselves needed to develop skills to manage both patients’ and partners’ needs and expectations, as those needs might be quite different from each other.

In terms of negative behaviours, HCPs emphasised that these were typically demonstrated by the partner rather than the patient. HCPs identified two characteristics of partners believed to impede adjustment by the couple: 1) partners who did not know *how* to provide support; and 2) partners who were viewed as not *wanting* to provide support (as perceived by the HCPs). HCPs commented that partners who did not know *how* to provide support would typically avoid discussions that might upset the patient, in an attempt to protect them from further distress, which in turn seemed to result in a lack of communication within the couple. HCPs described scenarios where couples’ reluctance to speak to each other about particular issues was often misinterpreted by each member of the couple, and as a result created a tension within the relationship.

*“[the couples who say] ‘I would talk to my partner about this*, *but they’re the patient now*, *they’re sick I can’t lay this on them’… then the other person thinks ‘why are they acting weird*?*’… it’s basically putting up a wall between you and them and then they just see the wall*.*”*

*(Psychologist)*

Based on their perceptions of partners’ behaviours during medical consultation, HCPs also provided examples of partners who simply did not *want* to provide support to the patient. HCPs often linked partners’ negative behaviours towards both the patient and the HCP to their level of emotional distress. Moreover, some HCPs expressed the view that such hostility was also indicative of pre-existing relationship problems, accentuated by the stress of cancer.

*“[Partners] can be quite angry or off put and just tend to sort of sit there with their arms crossed and they’re only there because they feel that they have to be there*. *And I guess that’s the sort of thing that stands out to me that they don’t really want to be there…”*

*(Oncologist)*

### What is Couple-focused Psychosocial Care?

“*What is Couple-focused Psychosocial Care*?” (Fig 1) is a theme that combines parts of “Psychosocial Care for One or Two?”, a theme identified in HCPs’ interviews as they described how best to support patients and partners, and elements of “Our Experiences with HCPs”, a theme that emerged from couples’ description of their positive and negative experiences with HCPs. Two sub-themes were identified for “*What is Couple-focused Psychosocial Care*”: 1) “The Value of Couple-focused Psychosocial Support”, 2) “What Support and Who Should Provide It?”.

#### The value of couple-focused psychosocial support

All HCPs, regardless of their discipline, identified the value of extending psychosocial support to include both patients and their partners as a means of improving quality of life. The perceived level of psychosocial care was recognised as a quality of care indicator. However, HCPs differed in terms of the specific value given to this type of care. Psycho-oncologists’ comments related to their perceptions of the communication skills couples needed to cope with cancer together, and emphasised that couple-focused psychosocial support can provide an otherwise unavailable platform to facilitate discussions about issues of concern to patients and partners and address communication problems. Communication was identified as having reciprocal value for patients and partners, in that they both benefited from discussing the impact the cancer was having on them, and assisted couples in maintaining a strong relationship in time of crisis.

*“One of the things I’ve noticed is that this [cancer] can have a really big impact on the way you relate to each other as partners…giving them that permission to say ‘yes*, *this is really awful*.*”*

*(Social worker)*

Of note, psycho-oncologists also identified that another value of couple-focused psychosocial support is an increased understanding among all HCPs regarding the particular needs and concerns of a couple, which in turn can be used to tailor support for patients and partners.

In contrast to psycho-oncology professionals, oncologists, and to a lesser extent nurses, typically felt that the value of couple-focused psychosocial support was in the specific benefits it has for patients and their recovery, and not so much the benefit for the partner. Nevertheless, oncologists often described that partners who had a greater understanding of what the patient was going through physically and emotionally typically provided better practical support to patients (e.g., adhering to medication; wound dressing).

*“I acknowledge that it’s important to manage the couple’s health as much as… not as much as but as an entity for the reason of it being helpful for the patient themselves*.*”*

*(Oncologist)*

Nurses held similar views to oncologists, insofar as suggesting anything (including psychosocial care) that is of benefit to the patient is potentially worthwhile, though at the same time indicated that it was often difficult to see the value in providing care of partners if it interrupted their already limited time with patients.

#### What support do couples need?

The two particular support needs of couples that HCPs identified were essentially extensions of individual needs: 1) the need for information; and 2) the need for validation of psychological distress. Oncologists in particular stressed the importance of disease and treatment-related information. Oncologists suggested that it often took time for couples to get past immediate thoughts that cancer is synonymous with death, and to develop an understanding of what the specific diagnosis means for the patient moving forward. Most HCPs, and predominantly oncologists, described the difficulty in providing a clear picture of the patient’s situation to couples, and were mindful of how much information they needed to deliver and at what time point.

*“…often the partners become quite teary and they’re fretful that this is going to be a life threatening illness*, *then as they get more and more information*, *they get more confident in what’s going on*.*”*

*(Oncologist)*

The second dimension of this theme is the validation of distress. This aspect was most clearly articulated by nurses and psycho-oncology professionals. Although nurses and psycho-oncology professionals held similar views on the importance of cancer information to oncologists, they also stressed the importance of balancing cancer information with an acknowledgment that patients and/or partners might experience anxiety or depression as a result of the cancer diagnosis, and might also encounter relationship issues (e.g., sexual functioning) that could increase distress. The role of psychosocial support was described by psycho-oncology professionals in terms of HCPs conveying an understanding of the couples’ distress. Most psycho-oncology professionals viewed validation of couples’ distress as being the first step in developing a support plan that included both patients and partners.

*“… patients are looking for lots of normalisation and validation… the fact they are anxious or worried*, *that it is OK*, *they’re not going crazy … it’s very similar for both of them really because partners are looking for that as well*.*”*

*(Psychologist)*

Of note, patients and partners also raised the need for informational support, but instead of focusing on the type and amount of information received, they spoke of *how* it was delivered, highlighting the importance of trust and confident in their HCPs.

*“I think you can have ‘information overload’*. *As long as I feel comfortable in knowing what's going to happen then I really don't want to know the bad side effects of the drug I'm taking … I'd rather go there knowing I've got to have it and I'm not scared about having it*.*”*

*(Patient*, *breast cancer)*

Couples also overlapped with HCPs in terms of the importance of gauging the “normality” of their distress, particularly in regards to the beneficial role of support groups. Most couples felt that they could manage their stressors on their own or within their social networks (e.g., family, friends), and more intense support, such as counselling, was perceived as unnecessary.

*“The attitude that I’ve got*, *I don’t feel that they could sort of tell me anything I don’t already know or don’t already have…we’re good support for one another and as I said*, *we’re positive about it*, *I mean disappointed too [about poor prognosis]*, *but positive*, *so I really don’t need [psychosocial support]…Maybe later on I don’t know*, *but if it gets to a stage where they give you a timeframe on [survival]*, *maybe then*.*”*

*(Patient*, *head and neck cancer)*

Few couples reported seeking additional psychosocial support beyond what was typically offered to them, which most often included cancer support groups, dedicated clinical care nurses, and cancer specific telephone support (mainly offered through the Cancer Council). Couples often felt that making social comparisons to other support group members helped them to accept their own situation, and that examples of survivorship from within the group helpfully re-frame their outlook as they moved through their treatment.

#### Who should provide couple-focused psychosocial support?

Differences emerged among HCPs regarding their views on who provides psychosocial care for couples. Psycho-oncology professionals were clear that when appropriate to do so (i.e., the patient wanted the partner to be involved), they preferred to provide psychosocial support to patients and partners together. Oncologists most often felt they lead the care of their patients including ensuring patients were appropriately referred to receive the psychosocial support they needed. A minority of oncologists acknowledged that efforts should be made to include partners in these referrals, but no oncologists discussed the development of a specific care plan that included the partner. Oncologists and nurses, despite recognising the key role of partners, were generally explicit that their immediate focus was the patient. Nurses and oncologists suggested that although they are often faced with distressed couples, they did not believe that providing psychological support was part of their role, and were either reluctant to engage with couples, or simply did not have the ability, time or resources to explore psychosocial issues with couples. Moreover, most oncologists and nurses described that once the need for additional support was identified (based on their own clinical judgement or that of another HCP), their main role with partners was to essentially facilitate access to supportive care services through referrals.

### “How Can Couple-Focused Psychosocial Care be Improved?”

“*How Can Couple-Focused Psychosocial Care be Improved*? combines elements from the sub-theme “Improving Psychosocial Support During Survivorship” (from the couples’ theme “Transition to Survivorship”) and the sub-theme “Provision of Information” (from the couples’ theme “Our Experiences with HCPs”; Table 5) and the themes “The Quest for Adequate Psychosocial Care” and “Issues in Distress Screening for Couples” (HCPs; Table 4); This theme reflected HCPs’ and couples’ perspectives on the two key elements related to couple-focused psychosocial care that could be improved: 1) provision of information and 2) addressing barriers to psychosocial care.

#### Provision of information

Provision of information was identified by both HCPs and couples as an area that could be improved. HCPs from all disciplines acknowledged that information was often misunderstood by patients and partners, and that improvements were needed to increase couples’ understanding and confidence when discussing their situation.

*“Once the people get into the treatment they sort of realise ‘Well I didn’t necessarily understand what you were saying*. *I’ve suddenly got these side effects and it was only 2% of the population that gets them*. *I’m that 2% and it’s bad*. *What are we going to do about that*?*’ So there are a lot of issues around understanding what’s been said and the responsibility for that falls both to the health care professional and the patient and their carer*.*”*

*(Nurse)*

Most HCPs described the difficult balancing act between tailoring the information they provide, and providing all available information. Provision of information was equally raised by couples as an area for improvement, particularly as it pertained to the prognosis and emotional consequences of the diagnosis and treatment. Most couples preferred HCPs to be completely transparent in the information provided about the disease and likely outcomes, and couples did not mention having discussions regarding information preferences with HCPs. Couples’ reports of negative experiences with HCPs typically related to a lack of consistency of information among HCPs, and a lack of clarity about their diagnosis and treatment. For partners, the lack of clarity of information contributed to the perceived burden of new care responsibilities, noting the use of technical language by HCPS and lack of clarity regarding prognosis.

*“We’d been seeing [oncologist]*, *but we hadn’t considered that it was incurable*. *We just assumed that it was curable*. *We hadn’t asked and maybe they’re the questions you’re afraid to ask…[partner] said “is it going to kill him*?*” and [oncologist] says “Yes”*. *We hadn’t considered it was that serious up until then…until he dropped that bomb that day…”*

*(Patient*, *head and neck cancer)*

Couples also identified the need for more information about the often unexpected emotional consequences of cancer and treatment course, particularly referring to experience of anxiety and depression, mood changes, and the sense of helplessness that can accompany prolonged exposure to a stressor such as cancer. Although couples often spoke of being buoyed by the confidence of their HCP (usually oncologists or nurses) in discussing the medical aspects of their cancer, they did not describe having oncologists and nurses having the same level of confidence discussing the psychological aspects.

*“There’s a lot of stuff that’s kind of kept in the background*. *You’re not told*, *like I said*, *his personality changed*. *I didn’t know it would change*.*”*

*(Partner*, *man with head and neck cancer)*

#### Improving HCP-perceived barriers to psychosocial care for couples

HCPs’ described several barriers to psychosocial care for couples that need to be addressed to improve couple-focused psychosocial care: 1) lack of acknowledgement and screening of partners’ distress, and 2) perception that couple-focused care is outside their expertise. Some HCPs felt that a lack of acknowledgment of partners’ distress and their practical concerns had a negative impact on couples facing cancer. In particular, psycho-oncologists described how partners often feel their distress is invalid, which can create additional distress within the couple.

*“Whoever is in front of the patient and the partner more often could say ‘And how are you going*?*’ to the partner… that validation could be the difference between the levels of distress… they’re still going to be very stressed*, *but one reason is just because they feel it’s invalid*.*”*

*(Psychologist)*

Psycho-oncologists felt that increasing understanding among HCPs of the interdependencies between partners’ and patients’ adjustment can start overcoming some barriers. On the other hand, social workers and nurses described often being patient-focused due to a lack of time, resources, and training to adequately address the patient’s needs, let alone the needs of both members of the couple.

*“If they’re a struggling sort of couple in any way then they should be dealt with by a counsellor as a couple*, *because as nurses we do our very best*, *but we’re not counsellors*, *[…] But apart from that we don’t have the time*.*”*

*(Nurse)*

Despite awareness of patients’ and partners’ distress, HCPs felt that the process of referring couples to receive psychosocial support lacked consistency within and between their respective health services. Views on current strategies for screening for emotional distress were central to this issue. The majority of HCPs believed that all patients and partners should be screened for distress, although given that the logistics of implementing standardised screening procedures for patients alone are often very difficult, it was acknowledged that this may not be realistic. Although most HCPs described the development of sensitive standardised screening tools was an important consideration, others felt that screening may bring additional problems to the surface that might create an additional burden for the couple or HCPs. Some preferred to rely on their own experience and the experience of other HCPs, believing that specific screening tools may not necessarily improve the detection of distressed individuals or couples.

*“I’m such a practical person…let’s get on with this and get this sorted and if we find a problem we’ll deal with it*. *Let’s not go delving*, *looking for problems…let’s not make problems where they may not exist*. *I think the screening tool is good and … I would never ignore a problem but… I’ve been a nurse for 45 years*, *I think I sense people’s emotions*.*”*

*(Nurse)*

Moreover, many HCPs felt that commonly used screening practices (e.g., pencil-and-paper measurement tools) were not capable of capturing either the reality of patients’ cancer experiences, or partners’ and couples’ experiences. In particular, the practice of screening at only one time point was viewed as reducing the likelihood of identifying distressed individuals at the time when they need help the most.

*“…The problem with that is that it’s usually done in a cross-sectional way without any sense of context…If it was longitudinal*, *I think there would be a much higher success rate of intervention*, *because it wouldn’t be so broad*, *it would be targeted*.*”*

*(Oncologist)*

## Discussion

With the increasing evidence that some couple-based interventions are efficacious in reducing distress and improving quality of life [2, 51], there is growing interest in developing models of psychosocial care in cancer that include both patients and their partners [52]. Nevertheless, implementation of couple-based interventions and approaches to psychosocial care for couples vary widely [30]. This study highlighted that whereas HCPs and couples perceived the value of couple-focused psychosocial support, they held very different perspectives as to how it should be implemented, which is consistent with previous studies with patients and HCPs [24, 26]. Specific points of divergence in previous studies mirrored the findings of the current study and included the role of partners [16], the provision of information [17, 19], who should deliver psychosocial care [25], and how distressed patients and partners are identified [28].

This study identified several themes and sub-themes that can be summarised within two categories of potential barriers and facilitators to the psychosocial care of couples: 1) person-level barriers, and 2) organisational-level barriers. Person-level barriers and facilitators have been categorised as the behaviour/s of a person (HCP, patient or partner) that might influence the provision of couple-focused psychosocial care [53]. Organisational-level barriers were categorised as the organisational policies, infrastructures, or behaviours of particular groups of people (e.g., HCPs from different disciplines) that influence the delivery of psychosocial care for couples [53].

One person-level barrier was related to divergent opinions regarding the perceived need for couple-focused psychosocial care. Most HCPs described couple-focused psychosocial support as being an important element of caring for patients and their partners, and described a preference for referring couples to specialist psychosocial services. On the other hand, most of couples felt that they did not need additional support, despite acknowledging that couple-focused care could be very valuable for those that required it. Referrals to separate HCPs for psychosocial support were thus seen as unnecessary by most couples, which suggests a preference for psychosocial care to be built in to routine care. This lack of perceived need for couple-focused assistance might be due to a limited awareness of what psychosocial support can encompass. For example, a study by Lambert and colleagues [30] that explored the feasibility of a self-directed couple-based intervention found that couples preferred to self-manage psychosocial concerns, but also needed some assistance to do this. This suggests that some initial discussion about psychosocial need and concerns, and strategies to manage them, might be beneficial for couples who did not perceive a need for specific psychosocial care. This speaks to the need to increase communication between HCPs and couples about what they can expect in terms of the emotional impact of cancer, and to discuss options for seeking additional support if it is needed.

A second person-level barrier that was identified across HCPs’ and couples’ pertained to HCPs’ views of the partner’s place in cancer care. Similar to Lindau and colleagues [54], this study highlighted that oncologists and nurses tended to focus on the patient and described being hesitant to discuss issues pertaining specifically to partners, let alone couple-level issues. In interviews with couples, this lack of engagement from HCPs contributed to a sense of hopelessness among partners. This is an issue that is common among previous studies, as partners and caregivers often perceive that their concerns are less valid or important, and at times they feel ignored by HCPs [20, 55–57]. Given most couples suggested that counselling was unnecessary, increasing all HCPs capacity to engage with patients *and* partners and to discuss their emotional concerns might be a more feasible way of introducing couple-focused psychosocial care and ensure it is more closely aligned with couples’ needs.

Some patterns were noted between HCPs from different discipline regarding the partner’s role: psycho-oncology professionals emphasised the need to support and improve partner’s emotional functioning and their ability to be an active participant in the patient’s cancer experience as being important to maintaining a healthy couple relationship. In contrast, oncologists and nurses acknowledged that partners are an important source of support for patients, but did not feel that actively engaging with partners was part of their role. Oncologists and nurses often prioritised more immediate elements of patient care over the psychosocial needs of the partner and the impact this had on the patient and the patient-partner relationship, which is consistent with previous studies [32, 33]. Although some HCPs were not always willing or able to engage with partners regarding their feelings or experiences, they were alert to some of the negative interactions between patients and partners that are consistent with what has been previously described as protective buffering [58] and demand-withdraw communication [59]. Such styles of interaction have been associated with greater psychological distress in patient and partners [60, 61]. It should be noted that although HCPs conceptualised some negative behaviour as being indicative of not *wanting* to provide support, they could also be considered symptomatic of not knowing *how* to provide support.

A third person-level barrier that emerged in interviews with couples and HCPs is the lack of consensus between couples and HCPs, and HCPs from different backgrounds, regarding the type of support that would be most beneficial for couples. Although previous studies have highlighted that couple-focused care can improve various supportive behaviours (e.g., coping [62], communication [63]), HCPs in the current study did not highlight specific couple support mechanisms, such as improving couples’ understanding of one another’s emotional experience, or describing techniques to provide more practical support to one another. Rather, oncologists and nurses tended to focus on providing medical and treatment information, whereas psycho-oncology professionals focused on validation of distress. These could be viewed as extensions of individual-level support. In contrast to what is typically viewed as important for couple-focused care in the psycho-oncology literature (e.g., relationship functioning, sexuality [2]), couples did not focus on increased support per se, rather they were more concerned with the trust and confidence in their HCPs, and felt that improving transparency in information provision could improve this sense of trust. Consistent with previous research [64], couples indicated that having oncologists or surgeons who confidently and clearly presented their medical treatment options typically eased their anxiety and increased their own confidence about what to expect. Couples reported less confidence in recommendations for psychosocial support from HCPs than other treatment recommendations. This is consistent with previous research suggesting patients receive significantly less advice about psychosocial issues than medical issues [65, 66]. Encouragement from HCPs to seek additional psychosocial support, particularly from oncologists, has been suggested by couples as a potential determinant of their decision to seek counselling or not [67], which in part speaks to the influence of HCPs’ prejudices towards psychosocial care [68]. Previous research suggests HCP-patient discussions of psychosocial concerns is associated with greater participation in counselling or other additional psychosocial support by patients [69]. Nevertheless, it is interesting to note also that concerns held by HCPs about exacerbating distress can inhibit attempts to explore emotional problems among patients [64]. Thus, improving HCPs confidence and skills in initiating discussion of the psychosocial and emotional impact of cancer, and exploring couples information and support needs is required; however, it may be necessary to address the stigma associated with psychosocial care for both couples and HCPs [70].

In addition to person-level barriers, HCPs identified organisational-level barriers. The most prominent one was lack of appropriate training in the recognition and management of psychosocial distress. Despite acknowledging the priority attached to recognising emotional distress as a “Vital Sign” [71], HCPs felt that typical distress screening practices were at odds with the way in which cancer care is currently provided (e.g., the lack of consultation time available). Although some HCPs described screening for distress in terms of asking patients and partners how they were managing, the majority discussed screening as being instrument-driven (i.e., needing to use a measurement tool). There is evidence that suggests that as many as 90% of eligible patients consent to distress screening when screening is administered by dedicated research staff [72, 73]. On the other hand, clinician-led screening has been shown to vary by discipline, with nurses more likely to use routine screening measures than doctors [28]. Despite studies suggesting that partners are often significantly more distressed than patients [74, 75], screening partners for distress has not been widely adopted. Given the difficulties raised by HCPs regarding screening programs for patients only, additional studies are needed to further examine how screening for partners can be embedded in to clinical practice.

Another organisational-level barrier identified by HCPs was a lack of appropriate training to manage the psychosocial issues couples face, which is consistent with other studies in this field [53]. Given the high prevalence [76, 77] and interdependence [78, 79] of distress between patients and partners, increasing HCPs’ capacity to engage with couples and validate their concerns earlier in the treatment phase may strengthen couples’ ability to manage cancer in the short- and long-term. Some studies have shown that improving HCPs’ communication skills can improve patients’ satisfaction with care [80] and that such skills can be developed through education [81, 82]. A meta-analysis of communication-based interventions for HCPs in oncology suggested that these types of interventions can have a moderate effect on improving HCPs’ skills [83]; however, translation to everyday practice is limited [84]. A comprehensive model of communication skills training has been developed by Kissane and colleagues [84], and participants have reported significant improvements in communicating effectively with patients and families. Communication skills training may also improve HCPs’ information provision by encouraging if HCPs discuss with couples how they would prefer to receive information [85].

Previous research has highlighted several barriers that limit the provision of psychosocial care to individuals from a patient-only perspective [10, 86, 87]. These barriers include difficulties in HCP-patient communication, a lack of appropriately trained HCPs, concerns regarding distress screening, issues with obtaining reimbursement for services from health insurance providers, stigma surround the use of psychosocial services, and transportation issues. The current study overlapped on several barriers, namely issues involving HCP-couple communication and HCPs’ ability to identify and manage couples’ distress. Thus, the barriers identified in the current study are not in themselves unique; however, they highlight a broadening scope in oncology that suggests partners are integral to patients’ care.

### Limitations

This study had a low consent rate (20.5%), which might reflect the variation in recruitment methods (i.e., eligible participants might have been more likely to provide consent if they were approached by the research team, rather than clinicians, or vice versa). However, given this is a qualitative study, the issues of generalisability is less of a concern than recruiting participants with key characteristics. The decision to interview couples simultaneously might have introduced a bias towards recruiting couples that are more cohesive and were potentially less likely to have experienced a negative impact on their relationship resulting from the cancer diagnosis. We also recognise that selection bias might have been introduced to the study given the recruitment of some HCPs that were known to researchers, and the inclusion of psycho-oncology professionals. The inclusion of psycho-oncology professionals provides a broader context to the issues couples face across the cancer trajectory, and highlights where implementation issues of psychosocial care for couples might occur. However, every effort was made to ensure methodological rigour was applied to the study. Another limitation is the possibility of social desirability bias. That is, some participants may have responded to questions in a manner they thought was consistent with the research aims.

### Conclusions and Implications

This study highlighted some of the issues couples face when one spouse is diagnosed with cancer, and steps that HCPs can undertake to improve the support to such couples. As more people live with the direct and indirect effects of cancer a focus on improving HCPs’ ability to engage with couples to improve their self-management is required. Two key areas are evident that require additional attention. First, improve HCPs ability to engage with partners, and explore the needs of the couple. Previous research has shown that poor communication with patients and partners is associated with psychosocial distress and lower satisfaction with care [88, 89]. As couples might not always want specific counselling services, enhancing other HCPs’ self-efficacy in discussing psychosocial issues could be of significant benefit to couples. Second, there is a need to continue the development of interventions for couples. The current evidence regarding couple-based interventions suggests they have a low to moderate impact on reducing psychological distress [2, 51]. Future couple-based interventions should look to build on the current focus of addressing psychosocial and relationship issues, and explore the possibilities of interventions that aim to improve communication with health care professionals [90] and enhance couples’ ability to advocate for patient and partner needs [91].
